# Long Non-coding RNA Expression Patterns in Stomach Adenocarcinoma Serve as an Indicator of Tumor Mutation Burden and Are Associated With Tumor-Infiltrating Lymphocytes and Microsatellite Instability

**DOI:** 10.3389/fcell.2021.618313

**Published:** 2021-02-12

**Authors:** Dongdong Yang, Jinling Yu, Bing Han, Yue Sun, Steven Mo, Jing Hu

**Affiliations:** ^1^Department of Surgical Oncology, The Fourth Affiliated Hospital of Harbin Medical University, Harbin, China; ^2^Department of General Practice, The Fourth Affiliated Hospital of Harbin Medical University, Harbin, China; ^3^Department of Internal Medical Oncology, Harbin Medical University Cancer Hospital, Harbin, China; ^4^YDILife Academy of Sciences, Nanning, China; ^5^Chinese Academy of Sciences Center for Excellence in Brain Science and Intelligence Technology, Shanghai, China

**Keywords:** lncRNAs, tumor immunity, stomach adenocarcinoma, the Cancer Genome Atlas, immune checkpoint molecules

## Abstract

Long non-coding RNAs (lncRNAs) are crucial in controlling important aspects of tumor immunity. However, whether the expression pattern of lncRNAs in stomach adenocarcinoma (STAD) reflects tumor immunity is not fully understood. We screened differentially expressed lncRNAs (DElncRNAs) between high and low tumor mutation burden (TMB) STAD samples. Using the least absolute shrinkage and selection operator method, 33 DElncRNAs were chosen to establish a lncRNA-based signature classifier for predicting TMB levels. The accuracy of the 33-lncRNA-based signature classifier was 0.970 in the training set and 0.950 in the test set, suggesting the expression patterns of the 33 lncRNAs may be an indicator of TMB in STAD. Survival analysis showed that a lower classifier index reflected better prognosis for STAD patients, and the index showed correlation with expression of immune checkpoint molecules (PD1, PDL1, and CTLA4), tumor-infiltrating lymphocytes, and microsatellite instability. In conclusion, STAD samples with different tumor mutation burdens have different lncRNA expression patterns. The 33-lncRNA-based signature classifier index may be an indicator of TMB and is associated expression of immune checkpoints, tumor-infiltrating lymphocytes, and microsatellite instability.

## Introduction

Stomach adenocarcinoma (STAD) accounts for 90% of gastric cancers (Hoffmann, 2015). Surgical treatment remains the optimal treatment for early-stage STAD, but most STAD patients are diagnosed in an advanced stage (Song et al., 2017). Development of targeted drugs has benefited many patients with specific genetic mutations (Shih et al., 2005; Sequist et al., 2007), but short-term drug resistance is still a limitation of this treatment (Nishio et al., 1999). Recently, immune checkpoint inhibitors (ICIs) showed promising effects against STAD (Atkins et al., 2017; Farina et al., 2017; Tosoni et al., 2017; Alsharedi and Katz, 2018; Killock, 2018; Li et al., 2018; Suresh et al., 2018; Togasaki et al., 2018; Arias Ron et al., 2019). However, ICIs are not effective for all STAD patients, so it would be helpful to identify indicators that can detect which patients are most likely to respond to ICIs.

Tumor immunogenicity, expression of immune checkpoint molecules and tumor-infiltrating lymphocytes are indicators of ICI treatment efficacy (Schreiber et al., 2011; Galon and Bruni, 2019). Tumor mutation burden (TMB), the number of somatic mutations in tumors as determined by whole-exon sequencing (Chalmers et al., 2017), is a widely used indicator of tumor immunogenicity and is also related to the therapeutic efficacy of ICIs (Alexandrov et al., 2013; Carbone et al., 2017; Hellmann et al., 2018). Efficacy of ICIs in STAD cancer patients is connected to high TMB levels (Kim et al., 2014; Goodman et al., 2019; Samstein et al., 2019; Wang et al., 2019). In patients with chemo-refractory advanced gastric cancer, overall survival rate was significantly higher in the high TMB group than in the low TMB group (Wang et al., 2019). However, the cost of determining TMB is high, and detection methods are not widely available in the clinic.

Long non-coding RNA (lncRNA) plays an important role in the development of various immune cells and the dynamic transcription process that controls markers that activate immune cells (Atianand et al., 2017). Under normal physiological conditions, expression of lncRNAs is tissue-specific and tightly regulated, whereas lncRNA expression in cancer is aberrant (Camacho et al., 2018). Therefore, we hypothesized that the expression pattern of lncRNA may be an indicator of TMB. We analyzed lncRNA expression patterns in low- and high-TMB STAD samples and defined a 33-lncRNA-based signature classifier that could accurately distinguish them. Our present may provide a cost-effective way to identify STAD patients more likely to respond to ICIs, thereby helping personalize treatment.

## Results

### LncRNAs Differentially Expressed Between High- or Low-TMB STAD Samples

**FIGURE 1:**
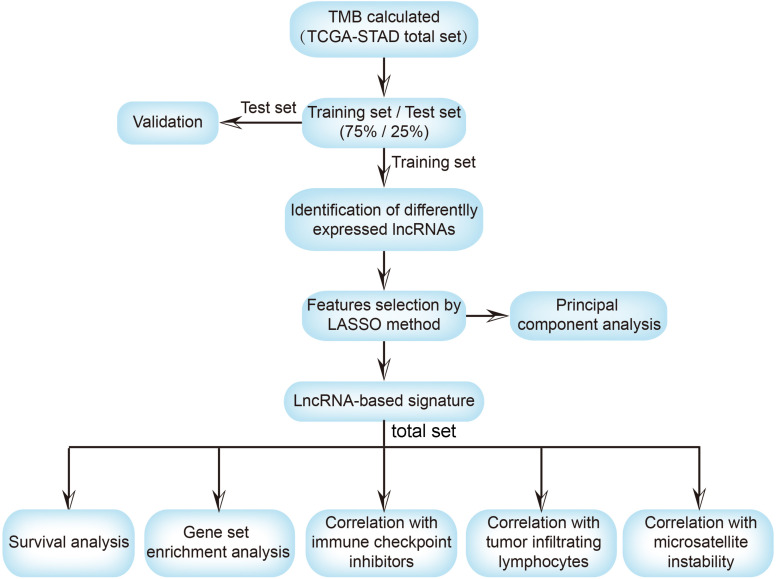
The workflow of this study. TMB, tumor mutational burden; TCGA, the cancer genome atlas; STAD, stomach adenocarcinoma; lncRNA, long non-coding RNA; LASSO, least absolute shrinkage and selection operator; ICI, immune checkpoint inhibitor; TILs, tumor-infiltrating lymphocytes.

**FIGURE 2:**
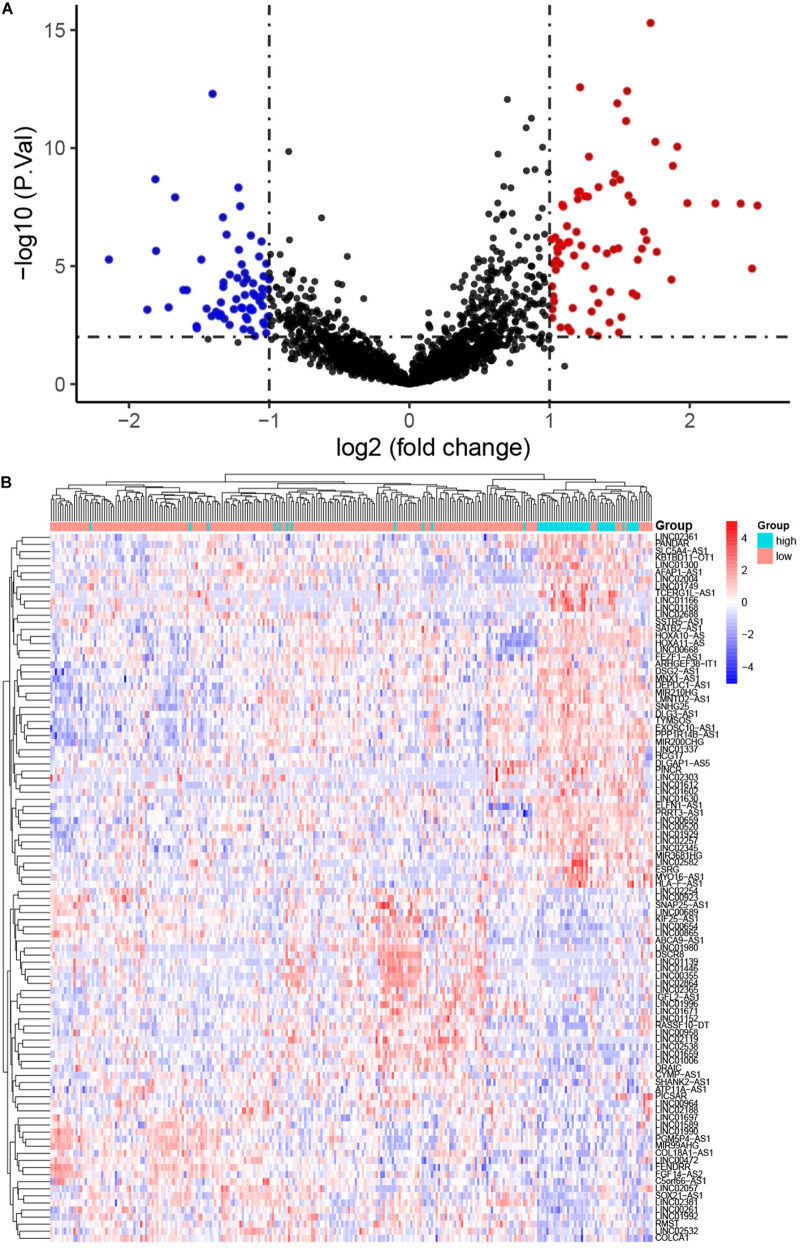
Differentially expressed long non-coding RNAs (DElncRNAs) between stomach adenocarcinoma (STAD) samples with high and low tumor mutation burden (TMB). **(A)** Volcano plot showing DElncRNAs. Red points represent up-regulated; blue points represent down-regulated RNAs; and black points represent no significant difference. **(B)** The expression pattern of DElncRNAs can basically distinguish the level of TMB in STAD.

The workflow of the present study is shown in Figure 1. We analyzed the lncRNA expression profiles and mutation data of 348 STAD samples, comprising 64 with high TMB and 284 with low TMB. These samples were randomly assigned to the training set (261 samples) and the test set (87 samples). The training set contained 48 high- and 213 low-TMB samples. There were no significant differences in routine clinicopathological characteristics between the training and test sets (Table 1). A total of 309 lncRNAs were differentially expressed in the high-TMB sample compared to low-TMB samples (Figure 2A). Among these differentially expressed lncRNAs (DElncRNAs), 205 were up-regulated, and 104 were down-regulated. Hierarchical clustering showed that expression of DElncRNAs could distinguish STAD samples with different levels of TMB (Figure 2B).

**TABLE 1:** Summary of patient cohort information.

Factor	Cohort		*P*-value
	Training (*N* = 261)	Test (*N* = 87)	
Gender			0.797
Male	165	57	
Female	96	30	
Age (year)			0.050
<65	117	27	
≥65	141	59	
Not available	3	1	
T			0.571
T1–2	65	25	
T3–4	196	62	
N			0.852
N0	79	30	
N1–3	177	56	
Nx	4	1	
Not available	1	0	
M			0.091
M0	235	73	
M1	17	6	
Mx	9	8	
Pathological stage			0.803
I–II	123	39	
III–VI	138	48	
Histological type			0.599
Diffuse type	45	13	
Mucinous type	16	3	
Not otherwise specified	141	49	
Papillary type	4	1	
Signet ring type	8	2	
Tubular type	47	18	
Not available	0	1	
Microsatellite			0.904
MSI-H	46	15	
MSS-L	41	12	
MSS	173	60	
Not available	1	0	
Grade			0.840
G1	5	3	
G2	93	30	
G3	157	52	
Gx	6	2	

*MSI-H, microsatellite instability-high; MSI-L, microsatellite instability-low; MSS, microsatellite stability.*

### The 33-LncRNA-Based Classifier Index

**FIGURE 3:**
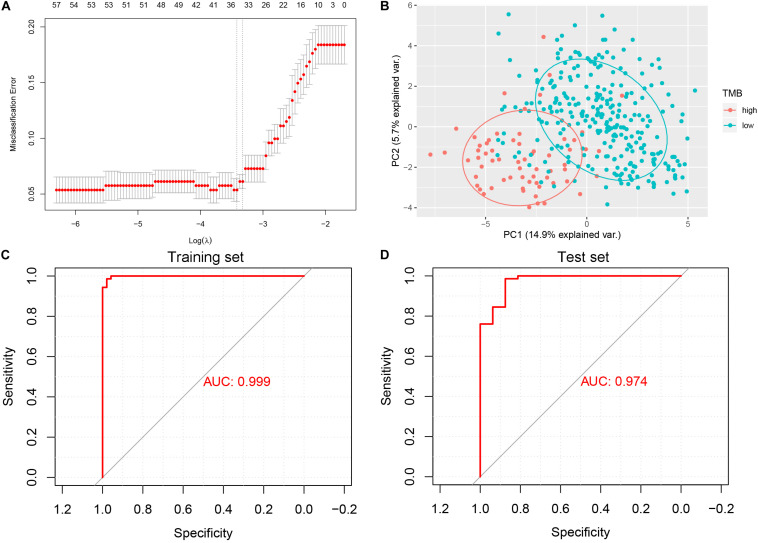
Least absolute shrinkage and selection operator (LASSO) and receiver operating characteristic curve analysis. **(A)** 10-fold cross-validation for tuning parameter selection in the LASSO model. **(B)** Scatter plot of the first and second principal components. **(C)** Receiver operating characteristic curve analysis in the training set. **(D)** Receiver operating characteristic curve analysis in the test set. AUC, area under the curve.

The least absolute shrinkage and selection operator (LASSO) logistic regression method developed a lncRNA-based classifier using the expression profiles of the DElncRNAs in the training set. A total of 33 DElncRNAs were identified as optimal lncRNAs with non-zero regression coefficients (Figure 3A). Principal component analysis demonstrated that the expression patterns of the 33 DElncRNAs could distinguish samples with high or low TMB (Figure 3B). The accuracy of the 33-lncRNA-based classifier was 0.970 in the training set and 0.950 in the test set, and the sample recognition efficiency of the classifier was high (Table 2). Additionally, receiver operating characteristic (ROC) curve analysis confirmed that the 33-lncRNA-based classifier could predict TMB levels of STAD samples, with an area under the curve (AUC) of 0.999 (Figure 3C) in the training set and 0.974 (Figure 3D) in the test set. Moreover, the difference in AUCs between the training and test set was not significant (*P* = 0.190). Higher values of the classifier index were associated with lower TMB.

**TABLE 2:** Performance of the 33-lncRNA-based classifier index in predicting tumor mutation burden (TMB) in stomach adenocarcinoma (STAD) samples.

Group	Se	Sp	PPV	NPV	Accuracy	AUC
Training set	0.830	0.960	1.000	0.960	0.970	0.999
Test set	0.560	0.910	1.000	0.910	0.950	0.974

*AUC, area under the receiver operating characteristic curve; NPV, negative predictive value; PPV, positive predictive value; Se, sensitivity; Sp, specificity.*

### Biological Characteristics of STAD With High LncRNA-Based Classifier Index

**FIGURE 4:**
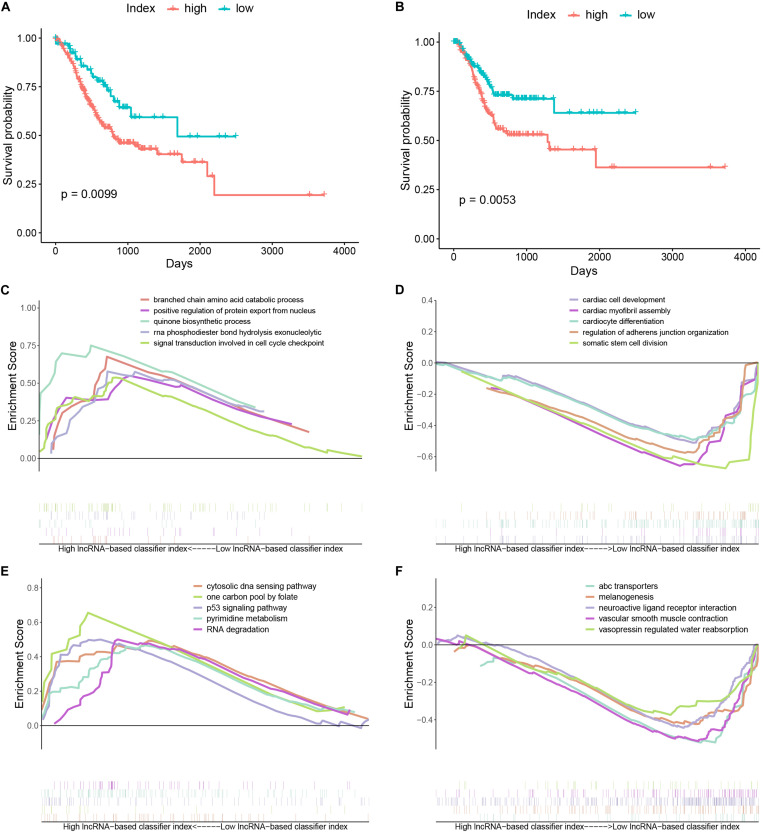
Kaplan–Meier curves and gene set enrichment analysis. **(A)** Kaplan–Meier curves of overall survival in the high- and low-index groups. **(B)** Kaplan–Meier curves of relapse-free survival in the high- and low-index groups. **(C)** Top 5 significant biological processes enriched in samples with a high value of the 33-lncRNAs-based classifier index. **(D)** Top 5 significant biological processes enriched in samples with a low value of the 33-lncRNAs-based classifier index. **(E)** Top 5 significant KEGG pathways enriched in samples with a high value of the 33-lncRNAs-based classifier index. **(F)** Top 5 significant KEGG pathways enriched in samples with a low value of the 33-lncRNAs-based classifier index.

Stomach adenocarcinoma (STAD) patients with a high lncRNA-based classifier index had worse overall survival (Figure 4A) and recurrence-free survival (Figure 4B). Biological processes (BPs) enriched in samples with a high lncRNA-based classifier index included branched chain amino acid catabolic process, positive regulation of protein export from nucleus, and signal transduction involved in cell cycle checkpoint (Figure 4C). Meanwhile, BPs enriched in samples with low values of the lncRNA-based classifier index were cardiac cell development, cardiac myofibril assembly, cardiocyte differentiation, and regulation of adherens junction organization (Figure 4D). KEGG pathways enriched in high-index groups included cytosolic DNA sensing pathway, p53 signaling pathway, and pyrimidine metabolism (Figure 4E), while those enriched in low-index groups were melanogenesis, neuroactive ligand receptor interaction, and vascular smooth muscle contraction (Figure 4F).

### The 33-LncRNA-Based Classifier Index Associate Expression of Immune Checkpoint

**FIGURE 5:**
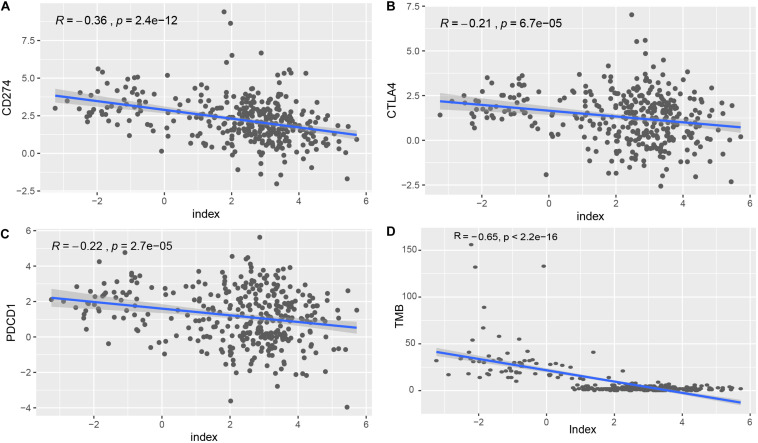
Correlation of the 33-lncRNA-based signature index with expression of ICIs index (CD274, CTLA4 and PDCD1) and TMB. **(A)** Correlation of the 33-lncRNA-based signature index with expression of CD274. **(B)** Correlation of the 33-lncRNA-based signature index with expression of CTLA4. **(C)** Correlation of the 33-lncRNA-based signature index with expression of PDCD1. **(D)** Correlation of the 33-lncRNA-based signature index with expression of TMB.

To explore the roles of the classifier index in STAD, we assessed correlations of the 33-lncRNA based classifier index with the expression of three immune checkpoint molecules (PDCD1 also known as PD1, CD274 also known as PDL1, and CTLA4). The 33-lncRNA based classifier index showed significant correlation with CD274 (Pearson *R* = -0.36, *P* = 2.4e-12), CTLA4 (Pearson *R* = -0.21, *P* = 6.7e-05) and PDCD1 (Pearson *R* = -0.22, *P* = 2.7e-05) (Figures 5A–C). Moreover, the 33-lncRNA based classifier index showed significant negative correlation with TMB (Figure 5D).

### The Correlation Between the 33-LncRNA-Based Classifier Index and Tumor Infiltrating Lymphocytes

**FIGURE 6:**
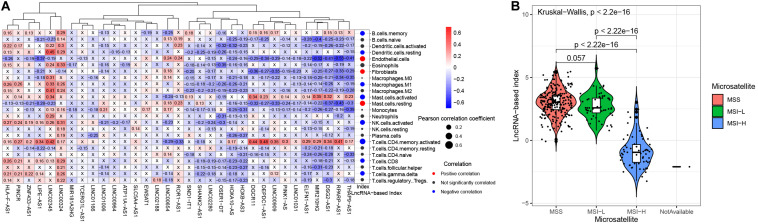
The associations between the 33-lncRNA-based classifier index and tumor infiltrating lymphocytes, and microsatellite status. **(A)** The correlations between the 33-lncRNA-based classifier index and tumor-infiltrating lymphocytes. **(B)** The 33-lncRNA-based classifier index in patients with different microsatellite status.

We explored the correlation of the 33 lncRNAs and tumor-infiltrating lymphocytes (Figure 6A). The lncRNA LINC02345 was positively correlated with resting dendritic cells, M2 macrophages and activated memory CD4 T cells. Activated memory CD4 T cells were also positively correlated with lncRNAs DGCR11, DEPDC1-AS1, LINC00909, MIR210HG, DSG2-AS1, and RPARP-AS. These most of these 33 lncRNAs were negatively correlated with endothelial cells, fibroblasts and resting mast cells. The 33-lncRNA based classifier index was positively correlated with endothelial cells, fibroblasts and resting mast cells. It was negatively correlated with the following cells: Memory B cells, resting dendritic cells, M1 macrophages, activated mast cells, activated NK cells, activated memory CD4 T cells, CD8 T cells, and gamma delta T cells.

### The LncRNA-Based Index Associates With Microsatellite Instability

Microsatellite instability-high (MSI-H) is associated with response to treatment of ICIs in STAD (Fuchs et al., 2018). We found that STAD patients with MSI-H have a lower lncRNA base index (Figure 6B) than those with MSI-low or microsatellite stable (MSS).

## Discussion

Tumor mutation burden (TMB) is used in clinical practice for predicting ICI treatment efficacy, and high TMB is related to better prognosis in patients treated with immunotherapy (Rizvi, 2018; Samstein, 2019). However, TMB detection is too expensive to be widely used in clinic. In the present study, we screened the DElncRNAs between STAD samples with high or low TMB, and identified a 33-lncRNA-based signature classifier that can assess TMB in a cost-effective manner. The accuracy of the 33-lncRNA-based classifier was high and robust. Moreover, the classifier had a high specificity, positive predictive value and negative predictive value. The expression pattern of these 33 lncRNAs may be a novel indicator for predicting ICI treatment efficacy.

Tumor mutation burden (TMB) is a robust predictor of the efficacy of ICI treatment (Cao et al., 2019) and a potential prognostic biomarker (Romero, 2019). We found that a low value of our 33-lncRNA-based index was associated with better overall and recurrence-free survival in STAD patients. Interestingly, immune-related biological pathways and KEGG pathways were not enriched in STAD samples regardless of the value of the lncRNA-based index. This may be because the molecular function of most lncRNAs is still unknown. The biological mechanism underlying these 33 lncRNAs associated with TMB levels required further exploration.

Tumor mutation burden (TMB), expression of immune checkpoint molecules and tumor-infiltrating lymphocytes are associated with ICI efficacy. However, TMB may predict the efficacy of ICI independently of the expression level of immune checkpoint molecule CD274 (Carbone et al., 2017). Our 33-lncRNA-based signature classifier index showed significant low correlation with PDCD1, CD274, and CTLA4. This suggests that the signature classifier index cannot replace the measurement of immune checkpoint expression for predicting ICI efficacy. ICIs appear to be more effective against tumors when they have been infiltrated by lymphocytes (Maleki Vareki, 2018). Our 33 lncRNAs were associated with numbers of resting dendritic cells, M1 macrophages, resting mast cells, activated NK cells and activated memory CD4 T cells, while the 33-lncRNA-based classifier index was mainly associated with numbers of memory B cells, resting dendritic cells, endothelial cells, fibroblasts, M1 macrophages, activated NK cells and activated memory CD4 T cells. In gastric cancer, high levels of tumor-infiltrating lymphocytes are related to better prognosis (Morihiro et al., 2019; Zhang et al., 2019). Moreover, STAD patients with MSI-H score have a lower lncRNA-based index. Thus, the 33-lncRNA-based signature classifier index is associated with four factors that can predict ICI efficacy: TMB, expression of immune checkpoint molecules, tumor-infiltrating lymphocytes, and microsatellite instability. Future prospective studies should investigate whether the index can be used in the clinic to predict ICI efficacy.

Although this study may provide an exciting potential surrogate measure for TMB in STAD, there are limitations worth mentioning. Firstly, the classifier needs to be validated in a larger independent data set. Second, the molecular mechanism of these 33 lncRNAs in the immune response is not yet clear. Thirdly, the relationship between the lncRNA-based index and other molecular typing systems were not clear.

In conclusion, STAD samples with different TMB levels have different lncRNA expression patterns. The 33-lncRNA-based signature classifier index may be an indicator of TMB and is associated expression of immune checkpoints, tumor-infiltrating lymphocytes, and microsatellite instability.

## Experimental Procedures

### Data Processing

The *GDCquery_Maf* function of the *TCGAbiolinks* package (Colaprico et al., 2016) was used to download STAD mutation annotation files from the Cancer Genome Atlas (TCGA) database^1^. The *MuTect2* pipeline (Cibulskis et al., 2013) called somatic mutations. The *read.maf* function in the *maftools* package (Mayakonda et al., 2018) downloaded maf data, which included variation samples and the variation count of each sample. In our study, TMB was defined as the number of somatic cell variations per megabase (MB) in the genome (Hellmann et al., 2018). High TMB was defined as ≥10 mutations/MB, and low TMB as <10 mutations/MB (Hellmann et al., 2018; Lv et al., 2019). The estimated size of the exome was 38 MB (Chalmers et al., 2017). The lncRNA and mRNA were identified according to the comprehensive gene annotation (version 33) file downloaded from gencode^2^. STAD stomach samples, with both lncRNA expression profiles and mutations, were randomly assigned to the training set (75%) and the test set (25%).

### Screening DElncRNAs in High-TMB Compared to Low-TMB STAD Samples

In the training set, the *voom* function (Law et al., 2014) was used to normalize the RNA sequence data of STAD. The *limma* package (Smyth, 2004; Law et al., 2014; Ritchie et al., 2015) was used to screen the DElncRNAs in high-TMB compared to low-TMB STAD samples. LncRNAs with a *P* (adjusted by the false discovery rate) < 0.01 (Lv et al., 2019) were considered significant. Based on Euclidean distance, we performed bidirectional hierarchical clustering (Szekely and Rizzo, 2005) using the expression profiles of DElncRNAs and displayed the results as a heat map using the *pheatmap* R package^3^.

### Feature Selection and Principal Component Analysis

Using the *glmnet* package (Friedman et al., 2010), LASSO logistic regression identified optimal lncRNAs with non-zero coefficient from DElncRNAs, and these lncRNAs were used to create a LASSO signature classifier. The corresponding parameters are set to “nfolds = 10” representing 10-fold cross-validation, and “family = ‘binomial”’ representing two-class logistic regression analysis. In addition, the expression profiles of the optimal DElncRNAs were used to perform principal component analysis. The first and second component was applied to visualize the result displayed as a two-dimensional scatter plot using *ggbiplot* package^4^.

### Construction of the LncRNA-Based Signature Classifier for Predicting TMB

Through LASSO logistic regression, we obtained the regression coefficient (Coef) of the lncRNA and calculated the classifier index for each STAD sample according to formula:

Index = Expr_*lncRNA*1_^∗^Coef_*lncRNA*1_ + Expr_*lncRNA*2_^∗^Coef_*lncRNA*2_ + Expr_*lncRNA*3_^∗^Coef_*lncRNA*3_ + ⋅⋅⋅ + Expr_*lncRNAn*_^∗^Coef_*lncRNAn*_

where “Expr” represents the expression value of the lncRNA. The classifier’s accuracy, positive predictive value, negative predictive value, sensitivity, specificity and area under the receiver operating characteristic curve (AUC) were determined. The receiver operating characteristic curves were generated using the *pROC* package (Robin et al., 2011) in R, and compared using the DeLong method (DeLong et al., 1988).

### Survival Analysis and Gene Set Enrichment Analysis

To further explore prognostic value of the lncRNA-based signature classifier index in STAD, survival analysis was performed using *survival* and *survminer* package^5^. The *surv_cutpoint* function in the *survminer* package was used to determine the optimal cutoff to divide patients high or low-index groups. GSEA2-2.2.4 (Java) version (Mootha et al., 2003; Subramanian et al., 2005) was used to explored potential biological characteristics in the high- or low-index groups. According to default parameters, the c5.bp.v6.2.symbols.gmt and c2.cp.kegg.v6.2.symbols.gmt datasets in MsigDB V6.2 database (Liberzon et al., 2015) were used as reference gene sets. Nominal *P* < 0.05 was considered significant.

### Correlation of the LncRNA-Based Signature Classifier Index With Expression of Immune Checkpoints

The expression of immune checkpoint molecules is another factor currently considered to affect ICI efficacy (Schreiber et al., 2011). Therefore, in our study, we investigated the correlation of the lncRNA-based signature classifier index with the expression of three immune checkpoints (PDCD1, CD274, and CTLA4). The correlation between the lncRNA-based signature classifier index and TMB was also explored.

### Estimation of the Correlations of Tumor-Infiltrating Lymphocytes and the LncRNA-Based Signature Classifier Index

Firstly, we integrated two gene signatures, CIBERSORT (Newman et al., 2015) and MCP-Counter (Becht et al., 2016), to identify the mark gene set of the tumor-infiltrating lymphocytes and stromal cells according to a previous study (Xiao et al., 2019). Subsequently, we used single sample gene set enrichment analysis to calculate the abundance of each cell subset in each sample based on gene expression data. Then, the pearson correlation between the lncRNA-based signature classifier index and the cell abundances were calculated. *P* < 0.05 was considered significant.

### Statistical Analysis

All the analyses were performed using R software^6^. The χ^2^-test was used for categorical data. An unpaired *t*-test was used to screen DElncRNAs. Kaplan–Meier survival curve analysis with log-rank method was used to compare survival between the two groups of patients. Kruskal-Wallis method was used compared the continuous variable between three groups. Unless otherwise stated, we considered *P* < 0.05 to be statistically significant.

## Data Availability Statement

The raw data supporting the conclusions of this article will be made available by the authors, without undue reservation.

## Author Contributions

DY, JY, SM, and BH performed the material preparation, data collection, and analysis. YS and JH wrote the first draft of the manuscript. All authors commented on previous versions of the manuscript, contributed to the study conception and design, read and approved the final manuscript.

## Conflict of Interest

SM was employed by the YDILife Academy of Sciences. The remaining authors declare that the research was conducted in the absence of any commercial or financial relationships that could be construed as a potential conflict of interest.

## Footnotes

1 https://gdc.cancer.gov/

2 https://www.gencodegenes.org

3 https://CRAN.R-project.org/package=pheatmap

4 https://github.com/vqv/ggbiplot

5 https://CRAN.R-project.org/package=survival

6 https://www.r-project.org/

## Supplementary Material

Supplementary Table 1 The 33 long non-coding RNA selected by LASSO method.
